# Analysis of six CYP450 genetic variants regarding the response to cannabidiol combined with anticonvulsant medication in mexican patients with drug-resistant epilepsy

**DOI:** 10.3389/fphar.2025.1626054

**Published:** 2025-08-21

**Authors:** Iris A. Feria-Romero, Luisa Rocha, Araceli Reyes-Cuayahuitl, Iris Martínez-Juárez, Daniel San-Juan, Consuelo Escamilla-Nuñez, Sandra Orozco-Suarez

**Affiliations:** ^1^ Unidad de Investigación Médica en Enfermedades Neurológicas, Hospital de Especialidades, “Dr. Bernardo Sepúlveda”, Centro Médico Nacional Siglo XXI, Instituto Mexicano del Seguro Social, Ciudad de México, Mexico; ^2^ Departamento de Farmacobiología, Centro de Investigación y Estudios Avanzados, del Instituto Politecnico Nacional, Tlalpan, Ciudad de México, México; ^3^ Servicio de Neurología Pediátrica, Hospital de Pediatría, Centro Médico Nacional Siglo XXI, Instituto Mexicano del Seguro Social, Ciudad de México, Mexico; ^4^ Epilepsy Clinic, Instituto Nacional de Neurología y Neurocirugía, Mexico City, Mexico; ^5^ Centro de Salud Poblacional, Instituto Nacional de Salud Pública, Ciudad de México, Mexico

**Keywords:** pharmacoresistant epilepsy, cannabidiol treatment, CYPs, SNP, antiseizure medication

## Abstract

**Background:**

Cannabidiol (CBD) reduces the frequency of seizures in individuals with specific epileptic syndromes, but its effectiveness for other types of drug-resistant epilepsy (DRE) is unclear. CYP450 enzymes primarily metabolize CBD. The aim of this study was to identify CYP450 genotypes regarding the response of CBD treatment concomitant with anti-seizure drugs in patients with DRE.

**Methods:**

This observational, retrospective, comparative study examined patients with DRE who incorporated CBD into their treatment. Genomic DNA was extracted from the patients’ blood. Patients were divided into two groups: CBD responders and CBD non-responders. Six genetic variants of CYP450 genes were analyzed using real-time polymerase chain reaction (PCR). Statistical significance was determined using Fisher’s exact, chi-squared, and Mann–Whitney *U* tests. The analysis of polymorphisms involved a statistical test for proportion differences of more than 10% between the comparison groups.

**Results:**

This study examined 47 patients with DRE, of which 68% showed a positive response to CBD treatment in combination with their current medications. Among the patients who did not respond to adjuvant treatment with CBD, the *CYP3A4* *1/rs2242480 genotype was present in 50%. These patients exhibited focal seizures along with various lesions in imaging studies. In contrast, the *CYP2B6* *1/*2 and *2/*2 genotypes were identified in 42% of patients with drug-resistant epilepsy who did respond to CBD treatment. These patients had unknown causes of their epilepsy and showed expected results in imaging studies.

**Conclusion:**

Treatment with CBD reduced seizures in most patients (68%), which was independent of etiology and seizure type. The genotype *CYP3A4* *1/rs2242480 may be associated with low response to CBD.

## 1 Introduction

About 20%–30% of patients with epilepsy are considered resistant to antiepileptic drugs (GBD-2016; Kitschen et al., 2023). Consequently, alternative therapies have been explored, including products derived from cannabis species, such as cannabidiol (CBD), which does not have the addictive properties of tetrahydrocannabinol (THC). Since 2018, the US Food and Drug Administration (FDA) has approved the use of a liquid formulation of CBD (100 mg/mL) in sesame oil (flavored). In combination with clobazam, it is indicated for the treatment of patients aged 2 years and older who have Lennox–Gastaut syndrome or Dravet syndrome. It is also prescribed to treat tuberous sclerosis complex with other epilepsy treatments in patients aged 2 years and older. These rare types of epilepsy begin in childhood and may last into adulthood (Golub and Reddy, 2021; Reddy, 2023).

Randomized clinical trials have shown the efficacy and safety of these drugs in children and adults who have seizures that are related to these conditions and are challenging to manage. Furthermore, non-randomized trials have also shown promising results for other types of epilepsy. However, the mechanisms of how these patients respond to the highly variable CBD are not entirely understood (Reddy, 2023). Research indicates that CBD has complicated pharmacokinetics and inconsistent bioavailability, and there is a lack of biomarkers for predicting its therapeutic effects.

The bioavailability of CBD varies greatly according to the route and mode of administration. In clinical trials and research studies, CBD has generally been administered orally in capsule form or dissolved in oily solutions. It can also be administered sublingually or intranasally. A wide range of oral doses has been reported in the literature, with most doses ranging from 10 mg/day to 800 mg/day (in high-dose cases) (Millar et al., 2020; Huestis, 2007; Fasinu et al., 2016). The absorption of CBD in the gastrointestinal tract is erratic, and the resulting pharmacokinetic profile is variable. These characteristics are probably due to the poor aqueous solubility of CBD, as well as the method of administration (food vs. beverage), co-administration with additional ingredients, and preparation (as a ready-made product, it is better than a powder that the consumer must mix with liquid before ingestion).

Once ingested, the circulating concentration of CBD is influenced by absorption rates in the gut, breakdown during first-pass metabolism, and potentially by the body size and composition of the consumer. The bioavailability from oral administration was estimated as 6% due to significant first-pass metabolism (Fasinu et al., 2016; Ujváry and Hanús, 2016). Further complications arise when patients combine CBD with other medications or nutrients that could potentially interact with it, which can limit its efficacy and possibly enhance its toxicity (Zendulka et al., 2016)

**FIGURE 1:**
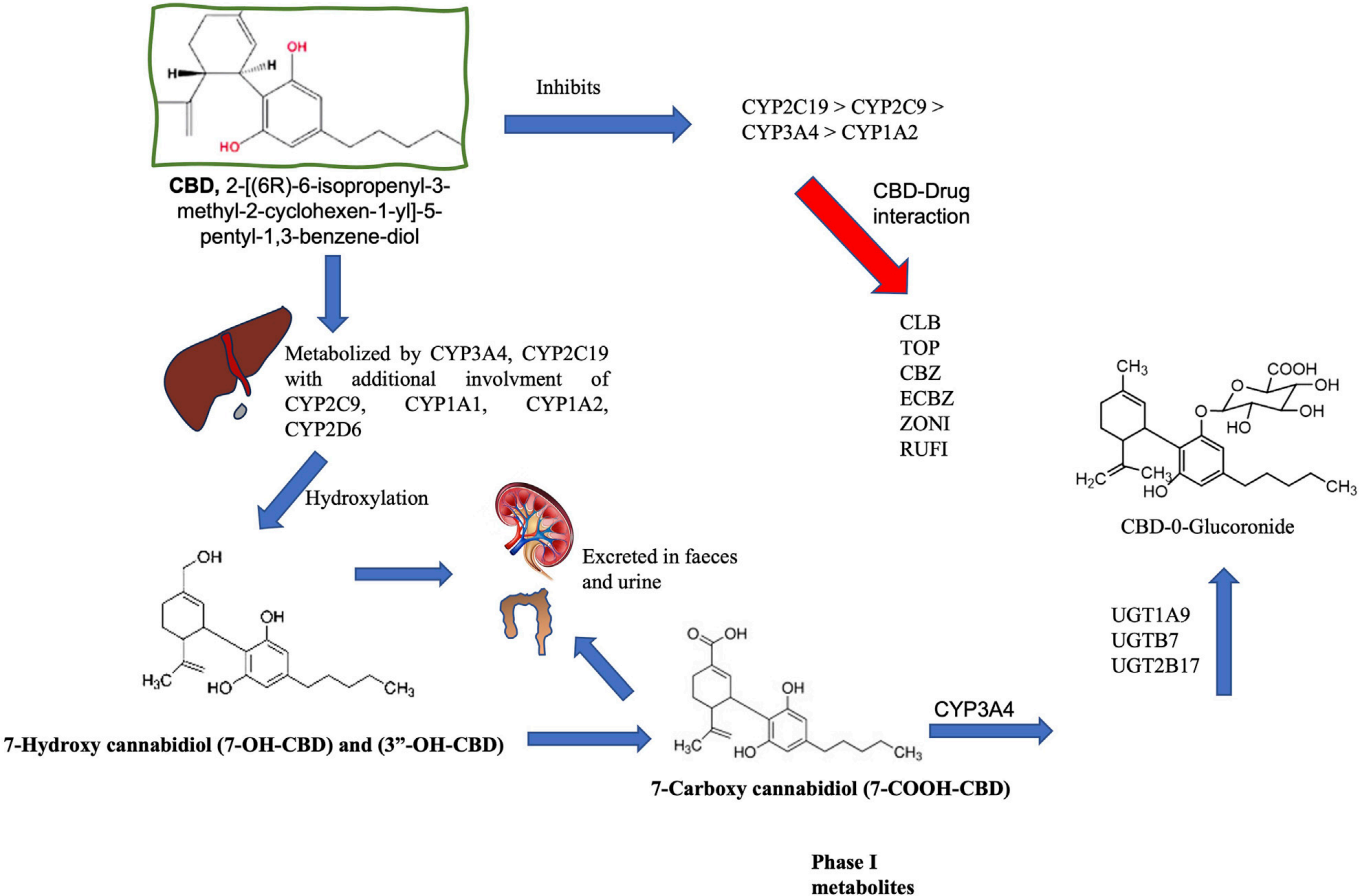
Metabolism of cannabidiol (CBD). CBD is metabolized by cytochrome P450 (CYP450) isoenzymes in the liver, and CYP3A4 and CYP2C19 are recognized as the primary phase I enzymes. CYP1A1, 2C19, 2D6, 3A4, and 3A5 also participate in biotransformation (CYP3A4 and CYP2C19 are the main phase I enzymes). These reactions initially oxidize the CBD molecule to produce 7-OH-CBD. CYP3A4 converts 7-OH-CBD into 7-COOH-CBD, a significant inactive compound in human blood. CYP3A4 is also involved in forming 6α-OH-CBD, 6β-OH-CBD, and 4-OH-CBD. This oxidation is followed by glucuronidation via UDP-glucuronosyltransferase (UGT) through enzymes such as UGT1A9, 2B7, and 2B17. Glucuronidation relies on prior processing by enzymes like CYP2C9 and CYP3A4, as well as the hydroxylated metabolites of CBD. The half-life of CBD in humans varies between studies (considering different doses and routes of administration) and can range from 1 h to 5 days. CBD is eliminated through metabolism and is excreted in an unchanged state, while metabolites are formed in urine and feces [Gonçalves et al., 2019, Harvey and Brown, 1991; Jiang et al., 2011; Ujvary and Hanus et al., 2016; reviewed by Chen et al., 2024]. Carbamazepine (CBZ), Clobazan (CLBZ), Eslicarbazepine (ECBZ), topiramate (TOP), Zonisamide (ZONI).

CBD is metabolized by cytochrome P450 isoenzymes (CYP450), particularly CYP2C19 and CYP3A4 and to a lesser extent CYP1A1, CYP1A2, CYP2C9, CYP2D6, and CYP3A5. The glucuronyltransferase activities of UDP-glucuronosyltransferase (UGT) enzymes UGT1A7, UGT1A9, and UGT2B7 also contribute (Harvey and Brown, 1991; reviewed by Chen et al., 2024). These reactions first oxidize the CBD molecule and form 7-OH-CBD, followed by other modifications that result in over 100 identifiable metabolites in several organisms (Jiang et al., 2011). CYP3A4 converts 7-OH-CBD into 7-COOH-CBD, a major inactive compound in human blood. Notably, CYP3A4 and CYP2C9 enzymes demonstrate higher affinity for CBD than other anti-seizure medication (ASM), which inhibit its metabolism by utilizing the same enzyme system (Figure 1; Harvey and Brown, 1991; Jiang et al., 2011; Ujvary and Hanus, 2016).

Previous reports have discussed how cannabinoids induce CYP450 enzymes to produce several metabolites. An *in vitro* study by Doohan et al. (2021) indicated that most cannabinoids did not affect CYP2D6 activity when dextromethorphan was used as a substrate. CBD partially inhibited CYP3A4 enzymatic activity when using triazolam, testosterone, or nifedipine as substrates. When observing CYP2C9-mediated tolbutamide metabolism, CBD caused more than 50% inhibition. CBD also impacted bupropion-mediated CYP2B6 activity, leading to an inhibition index (CI50) of 6.2 ± 1.7 μM. CBD also strongly inhibited CYPC19 activity, which is mediated by (S)-mephenytoin metabolism, with a CI50 of 2.1 ± 0.5 μM (Doohan et al., 2021).

As part of the drug-resistant epilepsy (DRE) hypothesis, pharmacokinetic theory suggests that the concentration of ASM entering the brain changes due to the unique expression of specific allelic variants of CYP450 metabolizing enzymes, which metabolize around 90% of all drugs (Tang et al., 2017). Genetic changes are associated with variations in drug responses, ranging from complete ineffectiveness to susceptibility to adverse drug reactions (Berger and Iyengar, 2011). In a study on pediatric patients with epilepsy, the single-nucleotide polymorphism (SNP) rs2242480 in *CYP3A4* (previously known as the *CYP3A4* *1G allele) was associated with drug responsiveness. This SNP was identified in 75% of drug responders and 43.5% of non-responders (Feria-Romero et al., 2023).

Thus, the aim of the present study was to determine whether six CYP variants are associated with response to CBD combined with ASM among pediatric and adult patients with DRE, to describe the benefit in seizure control when CBD is added to ASM, and to identify how this treatment is used in these populations.

## 2 Materials and methods

### 2.1 Patients and clinical information

The study, conducted between 2021 and 2024, was a retrospective, comparative analysis of Mexican pediatric and adult patients with drug-resistant epilepsy (DRE). These patients were seen at two leading neurological centers: the Pediatric Neurology Service of the Hospital de Pediatría, Centro Médico Nacional “Siglo XXI” of Instituto Mexicano del Seguro Social (IMSS), and the epilepsy clinic of the Instituto Nacional de Neurología y Neurocirugía “Manuel Velasco Suárez” (INNN). This study complied with the Mexican General Health and Helsinki Declaration principles. It received approval from the evaluation scientific and ethics committees, as denoted by registration numbers R-2019-785-008 and INNN-DI-IC-334-2021 from the IMSS National Commission and INNN, respectively. Adult patients with DRE and parents or guardians of severely disabled children or adults, who provided written informed consent for sample collection.

Response levels concomitant with ASM were evaluated after a minimum of 6 months of administering CBD treatment to DRE patients. DRE was defined as the inability to sustain a seizure-free state despite two tolerated and correctly administered ASM schedules, whether used alone or in combination (Kwan et al., 2010). Patients who did not respond to CBD were labeled CBDneg, while those who experienced a reduction in frequency were labeled as CBD^pos^.

The CBD dosage calculation was based on the concentration indicated by the patients for the product they used, calculated from the amount they consumed daily at any time. Of these patients, 10.25% used a homemade formulation, indicating that they were not using a commercial product but were aware of the CBD concentration. 7.6% used a commercial brand but were unaware of its concentration, and 82% used commercially produced CBD. The most commonly used commercial product has a concentration of 20.4 mg/mL.

During the evaluation period, the patients who were part of the protocol maintained their stable ASM treatment as it was before starting the CBD.

The database was created by collecting clinical, therapeutic, and genetic data from patients with DRE. Clinical variables included age, sex, etiology of epilepsy, type of seizures, therapy effectiveness, prior and current treatments, the number of simultaneous ASMs administered during follow-up, disease progression time, and results from electroencephalography and neuroimaging. We also included Latin American reference populations of European ancestry from the National Center for Biotechnology Information (NCBI) database to compare allele frequencies with those of a healthy population.

### 2.2 Sampling of genomic DNA

The blood sample was drawn into a BD Vacutainer^®^ tube. Each patient’s leucocytes were separated from blood samples or globular packages, using a Lysis RBC (Red Blood Cell) solution (# cat. PP-205S, Jena Bioscience DNA) through centrifugation. Following this, they were stored at −70°C. According to the supplier’s instructions, the patient’s genetic material was extracted from these packages using the Blood DNA Preparation kit (# cat.PP-205S, Jena Bioscience DNA). Finally, the extracted genomic DNA was kept at −70°C until further processing.

### 2.3 Identification of genetic variants

Purified DNA was analyzed using TapeStation and Genomic DNA ScreenTape Analysis from Agilent, and the concentration adjusted to 50 ng/μL. A real-time PCR reaction was conducted using 200 ng of genomic DNA, TaqMan SNP Genotyping Assays, and a corresponding probe, as per Thermo Fisher Scientific’s instructions. The reaction was performed on an AriaMx thermocycler (Agilent, United States) with the cycling parameters: 10 min at 25°C for one cycle, followed by 50 cycles of 15 s at 92°C and 90 s at 60°C. Genotyping analysis was conducted with Agilent AriaMx software, version 2.0. The polymorphism probes (Thermo Fisher Scientific, United States) and their respective base changes studied were rs2242480 (SNP ID: C__26201900_30, C>T) in *CYP3A4*; rs8192709 (SNP ID: C__2818162_20, C>T) and rs8192719 (SNP ID: C__22275631_10, C>T) in *CYP2B6*; rs1799853 (SNP ID: C__25625805_10, C>T) in *CYP2C9*; rs3892097 (C__27102431_D0, C>T) and rs1065852 (C__11484460_40, A>G) in *CYP2D6*. The frequencies of the selected variants’ altered alleles (Alt alleles) were obtained from the GenBank database (https://www.ncbi.nlm.nih.gov/snp).

### 2.4 Statistical analysis

Qualitative variables were represented using descriptive statistics as simple frequencies and percentages. Fisher’s exact or Chi-square (one-sided) tests were used to determine the association between categorical variables. Furthermore, median differences were analyzed using the nonparametric Mann-Whitney U test, and multiple linear regression was performed using the CBD response and age as variables with onset and frequency of seizures, dosages, as covariable (GraphPad Prism Software v9.0 for Mac, San Diego, CA, United States). A p < 0.05 was considered significant. For the analysis of polymorphisms between CBD^pos^ (responders) and CBD^neg^ (non-responders), we used the statistical test of proportion difference (prtesti) with STATA v. 14.0, setting a criterion of a difference greater than 10% between the comparison groups.

## 3 Results

### 3.1 Characteristics of the study population

The 47 patients included in this study comprised 18 pediatric patients (38.29%) and 29 adult patients (61.70%) with DRE. The adult patients had a better response to CBD (51%) than pediatric patients (17%). Only patients who had used CBD treatment for over 6 months were considered.

All patients received it orally in liquid drop form (both adults and children). There were fewer non-responders to CBD treatment (32%) than responders (68%). Among the non-responders, seizure frequency remained unchanged or increased in some cases (3 of 15 patients; 20%). In addition, some patients reported adverse effects of ASM before CBD treatment, particularly with oxcarbazepine (4.5%). Furthermore, 8.5% of patients reported mostly drowsiness as an adverse effect of CBD. CBD was used in combination with mainly levetiracetam and valproic acid, which were the most used drugs in both groups (Table 1).

**TABLE 1:** Anti-seizure drugs used in combination with CBD.

ASM	CBD^neg^ (%) number of patients (%)	CBD^pos^ (%) number of patients (%)	AE (ASM,%)	AE (ASM + CBD, %)
TOP	3 (20)	7 (21.8)	2.12	4.25
CLBZ	2 (13.3)	5 (15.6)		4.16
VPA	**5 (33.3)**	**16 (50)**		8.5
LEV	**12 (80)**	**21 (65.6)**		8.5
VIG	1 (6.6)	1 (3.12)		
CLONA	3 (20)	7 (21.8)		
OXCA	3 (20)	6 (18.7)	4.25	4.25
LACO	3 (20)	1 (3.12)		
LAMOT	2 (13.3)	8 (25)	2.12	6.3
DFH	2 (13.3)	3 (9.3)	2.12	
PB	1 (6.6)	0		
PRIM	1 (6.6)	0		

Antiseizure medication (ASM), Topiramate (TOP), Clobazan (CLBZ), Valproic acid (VPA), Levetiracetam (LEV), Vigabratine (VIG), Clonazepam (CLONA), Oxcarbazepine (OXCA), Lamotrigine (LAMOT), Lacosamide (LACO), Phenytoin (DFH), Phenobarbital (PB), Primidone (PRIM). Adverse events (AE), Cannabidiol (CBD). The bold letters indicate the most used drugs in percentage terms.

While seizures were not eliminated in the responder group, the monthly seizure frequency was reduced by more than 50%. The sex distribution showed a higher proportion of males than females, and the participants’ ages ranged from 3 to over 50 years old. Focal seizures were most common in both the non-responder and responder groups. The origin of epilepsy in both groups was similar (structural or unknown origin) with no significant differences, which made the study group more homogeneous in this variable. Most patients in both groups used more than three ASM in combination with CBD. Neuroimaging studies revealed similar ratios of normal to structural magnetic resonance images in both groups (Table 2).

**TABLE 2:** Gender, age, epilepsy etiology, type and frequency of seizures, number of antiepileptic drugs, and findings in the imaging study of Mexican patients with DRE treated with CBD (N = 47).

	Patients with DRE, ASM and CBD		Statistical tests
	CBD^neg^ n = 15	CBD^pos^ n = 32	
Gender			χ^2^ = 0.511
Male:Female	12:3	22:10	df = 1 P value = 0.47
Age (years)			
Min – Max	0.4–59	3–54	M-W-U = 164.5
Median [p25 – p75]	15 [5.0–30.0]	33 [11.5–30]	P value = 0.085
Etiology of epilepsy			
Unknown cause, n (%)	6 (40)	12 (40)	χ^2^ = 0.5250
Structural, n (%)	7 (46.6)	13 (43.3)	df = 3
Syndrome, n (%) Genetic, n (%)	2 (13.3)	4 (13.3) 1 (3.33)	P value = 0.913
Type of seizure			
Focal onset, n (%)	9 (60)	12 (37.5)	χ^2^ = 3.67
Generalized, n (%)	5 (33.3)	11 (34.3)	df = 2
Both, n (%)	1 (6.6)	9 (28.2)	P value = 0.159
Frequency of seizures (seizures/month)			
Min - Max	2–180	0–30	M-W U = 32.5
Median [p25 – p75]	60 [12–100]	4.5 [0–6]	P value **< 0.0001**
Onset seizures (years with seizures)			
Min-Max	0.4–29.7	2-45	M-W-U = 131
Median [p25-p75]	5 [ 2-14]	12.0 [5.0-45]	P value < 0.053
Drugs administered other of CBD			
One drug, n (%)	--	5 (15.6)	χ^2^ = 6.05
Two drugs, n (%)	1 (6.6)	9 (28.12)	df = 4
Three drugs, n (%)	8 (53.3)	9 (28.12)	P value = 0.195
Four drugs, n (%)	5 (33.3)	8 (25)	
Five drugs, n (%)	1 (6.6)	1 (3.12)	
Min-Max	15.7–206.4	3.6–309	M-W U = 110
CBD (mean ± SD, mg)	90.81 ± 69.7	53.0 ± 63	P value < 0.09
Finding in the study of image (MRI)			
Normal, n (%)	7 (46.67)	18 (56.3)	χ^2^ = 0.4018
Structural lesions, n (%)	8 (56.33)	14 (43.3)	d = 1 P value = 0.52

Mann-Whitney U test: M-W-U; Chi-square: χ^2^. The bold letters indicate the most used drugs in percentage terms.

The results of multiple linear regression using CBD response as a variable, with the other covariables noted in the methods, were only significant for seizure frequency (p < 0.0001). When age was used as a variable, the only significant covariables were the dosage (p < 0.03) and onset of seizure (p < 0.02).

### 3.2 Analysis of genetic variants

We compared the genetic variants between a Latin-American population with European ancestry (LA2) and our study population. The results showed that only the SNP rs8192709 was significantly associated with the type of population. Specifically, there was a significant difference of 46% (*p <* 0.0001) between the Alt Allele (T) in the study population (48.5%) and LA2 (2.8%).

The genetic variants associated with the response or non-response to CBD adjuvant in combination with anti-seizure medication were analyzed. The analysis was performed to compare the proportions between genotypes with the genetic variant (He+Ho mut) in the non-responder (CBD^neg^) and responder (CBD^pos^) groups. They were then grouped by etiology (unknown, structural, and syndrome), seizure type (focal, generalized, and combined), and imaging findings (normal and lesion), as depicted in Table 3. None of the genetic variants presented significant differences in this analysis. However, a trend in the prevalence of each polymorphism was identified in the group of patients with DRE who responded or did not respond to adjuvant treatment with CBD, as well as in a subgroup of patients with specific clinical characteristics.

**TABLE 3:** CYP genes, SNPs, genotypes, location, comparison groups and subgroups and their clinical significance in Mexican DRE patients with ASM concomitant with CBD treatment.

Gene	SNP	Genotype/Allele^†^	Location^#^	Population sample	Subgroup	p	^#^Clinical significance
*CYP3A4*	rs2242480	He^#^	^ψ^NC_000007.14:g.99763843C>T	CBD^neg^ vs. CBD^pos^		**0.6134**	Not Reported
				CBD^neg^ vs. CBD^pos^	Etiology-Unknown	0.7737	
					Etiology-Structural	0.4813	
					Seizures- Focal	0.1767	
					RMI-Normal	0.5213	
					RMI-Lesion	0.2613	
*CYP2B6*	rs8192709	*1/*2 + *2/*2	^&^NP_000758.1:p.Arg22Cys	CBD^neg^ vs. CBD^pos^		--	Not Reported
				CBD^neg^ vs. CBD^pos^	Etiology-Unknown	0.6396	
					Seizures- Focal	0.7228	
					Seizures- Generalized	0.7084	
					RMI-Normal	0.6306	
	rs8192719	Ho_mut_ + He^#^	^ψ^NC_000019.10:g.41012868C>T	CBD^neg^ vs. CBD^pos^		**0.3348**	Not Reported
				CBD^neg^ vs. CBD^pos^	Etiology-Unknown	0.5574	
					RMI-Normal	0.4046	
					RMI-Lesion	0.7128	
*CYP2C9*	rs1799853	*1/*2 + *2/*2	^&^NP_000762.2:p.Arg144Cys	CBD^neg^ vs. CBD^pos^		**0.6675**	Drug-Response
				CBD^neg^ vs. CBD^pos^	Etiology-Structural	0.6745	
					Seizures- Focal	0.5403	
*CYP2D6*	rs1065852	*4 and*10	§NP_001020332.2:p.Pro34Ser	CBD^neg^ vs. CBD^pos^		**0.5755**	Drug-Response

†: https://www.pharmgkb.org/

#: no designated allele.

and: 3′UTR, region; §: Exonic region; ψ: Intronic region; % Splice Acceptor Variant.

#: https://www.ncbi.nlm.nih.gov/snp/rs10497275#clinical_significance

CBD^neg^, patients not improve their seizures or presented adverse effects with CBD; CBD^pos^, patients improved their epileptic seizures with CBD; SNP, single nucleotide polymorphism.

Statistical test for comparing proportions (prtesti, STATA v, 14.0).

The numbers in bold represent the statistical differences between the two study groups, CBD^pos^ and CBD^neg^.

#### 3.2.1 SNP rs2242480 in *CYP3A4*

The most significant differences were found in patients with focal seizures in the polymorphism analysis of SNP rs2242480 in *CYP3A4*. The prevalence of CT+TT genotypes was 64% for those without response to CBD treatment (CBD^neg^), while the prevalence was 33% for patients with a CBD treatment response (CBD^pos^), resulting in a difference of 31%. In the subgroup of patients with structural lesions, the prevalence of CT+TT genotypes was 57% for CBD^neg^ and 30% for CBD^pos^, resulting in a difference of 27. Therefore, these CT+TT genotypes were present in 50% of patients with drug-resistant epilepsy who did not respond to concomitant CBD treatment, had focal seizures, and exhibited lesions in their imaging studies.

#### 3.2.2 SNP rs8192709 in *CYP2B6*

The most significant differences regarding SNP rs8192709 in *CYP2B6* were observed in two specific subgroups of patients with epilepsy of unknown etiology. In the CBD^neg^ subgroup, the prevalence of the CG genotype was 27%, while in the CBD^pos^ subgroup, it was 42%, resulting in a difference of 15%. Similarly, in the subgroup of patients who exhibited normal imaging results, the CG genotype prevalence in CBD^neg^ was 36%, while it was 50% in CBD^pos^, resulting in a 14% difference. Consequently, this polymorphism, which was identified as a heterozygous genotype (CG), was found in 42% of patients with drug-resistant epilepsy who responded positively to CBD concomitant treatment, particularly those with unknown etiology and normal imaging studies.

#### 3.2.3 SNP rs8192719 in *CYP2B6*

The most significant difference regarding SNP rs8192719 in *CYP2B6* was identified in the CBD^pos^ group, where the prevalence of the CT+TT genotypes was 65%, while in the CBD^neg^ group, it was 43%, resulting in a difference of 22%. In the subgroup of patients with epilepsy of unknown etiology, the prevalence of CT+TT genotypes was 14% in the CBD^neg^ group and 35% in the CBD^pos^ group, leading to a difference of 21%. For patients with normal imaging studies, the prevalence of CT+TT genotypes was 14% of the CBD^neg^ group and 45% in the CBD^pos^ group, resulting in a difference of 31%. Overall, this polymorphism, either as a heterozygous or homozygous mutated genotype, was identified in 35% of patients with drug-resistant epilepsy who responded to CBD as concomitant treatment, particularly among those with unknown etiology and normal imaging results.

#### 3.2.4 SNP rs1799853 in *CYP2C9*

The most significant difference regarding SNP rs1799853 in *CYP2C9* was identified in the CBD^neg^ group, where the prevalence of the CT+TT genotype was 29%, while it was 17% in the CBD^pos^ group, resulting in a difference of 12%. Similarly, in the group of patients with structural etiology, the CT+TT prevalence was 21% in the CBD^neg^ group versus 3% in the CBD^pos^ group, with a difference of 18%. In the focal seizure subgroup, the CT+TT prevalence was 29% in the CBD^neg^ group compared to 10% in the CBD^pos^ group, leading to a difference of 19%. Overall, this polymorphism, whether as a heterozygous or homozygous mutated genotype, was present in 21% of patients with drug-resistant epilepsy who were unresponsive to CBD concomitant treatment, particularly those with structural etiology and focal seizures.

#### 3.2.5 SNP rs1065852 in *CYP2D6*

The most significant difference regarding SNP rs1065852 in *CYP2D6* was found in the CBD^pos^ group. For the CBD^neg^ group, the prevalence of the CT+TT genotypes was 14%, while in the CBD^pos^ group, it was 34%, resulting in a difference of 20%. In the subgroup of patients with unknown etiology, the prevalence of the CT+TT genotypes was absent (0% prevalence) for the CBD^neg^ group and 16% for the CBD^pos^ group, indicating a difference of 16%. In the subgroup of patients with normal imaging, the prevalence was 0% for CBD^neg^ and 19% for CBD^pos^, yielding a difference of 19%. Overall, this polymorphism, whether as a heterozygous or homozygous mutated genotype, was present in 16% of patients with drug-resistant epilepsy who responded to CBD concomitant treatment, particularly among those with unknown etiology and normal imaging studies.

#### 3.2.6 SNP rs3892097 in *CYP2D6*

The most significant differences regarding SNP rs3892097 in *CYP2D6* were observed in the CBD^pos^ group. The CT genotype in the CBD^neg^ group was absent (0% prevalence), but in the CBD^pos^ group, its prevalence was 14%. A notable difference of 10% was found in the structural etiology subgroup with a CT genotype prevalence of 0% in the CBD^neg^ group and 10% in the CBD^pos^ group. Consequently, this polymorphism, which is characterized by a heterozygous genotype, was present in 10% of patients with drug-resistant epilepsy who responded to CBD concomitant treatment and had a structural etiology.

## 4 Discussion

Pharmacogenetics is a factor that can influence the relative contribution of specific CYP450 enzymes to a drug-metabolism reaction. May result in significant interindividual and interethnic variability in the metabolism, disposition, and clinical response of various therapeutic agents (Tan et al., 2010). When a new drug is introduced, it is important to identify its response in the population. Therefore, the aim of this study has been to identify the genetic polymorphisms in CYP450 and assess their correlation with the anti-seizure drug metabolism. The results could help to explain the response to CBD, which was investigated among Mexican adults and pediatric patients with DRE from two hospitals. Our cohort was small due to difficulties in patient access to CBD and adherence to a treatment plan of at least 6 months, which were part of our inclusion criteria.

In addition to distinguishing drug-resistant patients from those responsive to CBD and other medications, we also investigated the most common genetic variations in Mexican pediatric patients with DRE, as previously identified in another study (Feria-Romero et al., 2023). We found no significant difference regarding seizure types between those who responded to the treatment and those who did not. This includes 2 cases of Lennox–Gastaut syndrome (4.25%), where there was no response, as well as 4 cases (8.5%) with juvenile myoclonic epilepsy (JME), which responded to the treatment. This suggests that the seizure type does not necessarily dictate the variability in the CBD treatment response.

Some syndromes appear to be more responsive to CBD treatment. This was shown in a randomized double-blind clinical trial involving 120 children and young adults with Dravet syndrome and drug-resistant seizures. That study demonstrated that oral administration of CBD (20 mg/kg/day) concomitantly with already established pharmacotherapy decreased seizure frequency compared to a placebo, but it increased the incidence of adverse effects (diarrhea, vomiting, fatigue, pyrexia, somnolence, and changes in liver function). The same study highlighted that the most frequently co-administered drugs were clobazam (65%), valproic acid/valproate (59%), stiripentol (42%), levetiracetam (28%), and topiramate (26%) (Lopera et al., 2022).

In the population included in this study, the most commonly used drugs in both groups were levetiracetam, valproic acid, and lamotrigine (Table 2). In combination with CBD, the adverse events reported were minimal, and the best responses were observed. It should be noted that CBD is also administered to patients who are treated with medications that have their own side-effect profiles. Co-administration increases the possibility of overlapping profiles through metabolic or transport pathways (Brown and Winterstein, 2019).

Regarding the type of seizure, patients with focal seizures were the least responsive to CBD (60%). In the case of CBD^pos^, patients with the 3 types of seizures (focal, generalized, and both origins) were the most responsive. In contrast, a separate report on adult patients with drug-resistant focal epilepsy found that 87% experienced a reduction in seizures by over 50% (Kochen et al., 2023). In comparison, 11% with focal seizures had no effect from CBD. In other research, an average seizure reduction of 67.8% was observed in a sample of 78 patients (median age 24 years) with various structural and genetic conditions who were receiving an average of three ASM treatments and 14 months of CBD. Furthermore, 68.8% of this group experienced a reduction in seizure frequency greater than 50%, while 11.5% became seizure-free (Espinosa-Jovel et al., 2023).

Another study on epileptic encephalopathies and cases of focal or multifocal epilepsy found that 29.4% of patients with encephalopathies and 22.7% of patients with focal or combined epilepsy responded positively to CBD (Perriguey et al., 2024). When comparing these studies to ours, it is clear that CBD can offer advantageous effects for several types of DRE, particularly in adult patients. Nevertheless, only a select few studies have reported patients becoming seizure-free after adding CBD treatment to their regimen (Espinosa-Jovel et al., 2023; Perriguey et al., 2024; Kochen et al., 2023).

The SNP rs2242480 is located in the intronic region C99763843T on chromosome 7 and was initially assigned the *CYP3A4* *1G allele. The Pharmacogene Variation Consortium (www.pharmvar.org) reassigned it as allele *CYP3A4* *36 to align with the official terminology before removing it from their list. Current studies regarding this variant refer solely to the SNP. This variant had the most notable difference between CBD^neg^ and CBD^pos^, but the difference was not significant. However, its prevalence was higher in these patients than in drug-resistant (30%) and controlled (33%) patients in a previous study (Feria Romero et al., 2023).

Previous research has linked this SNP with varying levels of drug efficacy and toxicity, and it has been associated with poor response to carbamazepine (CBZ) in patients with epilepsy (Zhao et al., 2021). A meta-analysis of the rs2242480 polymorphism in *CYP3A4* showed significant associations with the plasma CBZ concentration in the co-dominant heterozygous model (AG vs. GG, standardized mean difference (SMD) = −0.24, 95% confidence interval (CI) = −0.4 to −0.09, *P =* 0.002), dominant model (AA + AG vs. GG, SMD = −0.21, 95% CI = −0.36 to −0.06, *P =* 0.007), and overdominant model (AA + AG vs. CI = −0.36 to −0.06, *P =* 0.007). The results indicate that the G allele of this SNP could decrease the plasma CBZ concentration in cases of epilepsy (Zhang et al., 2021).

In contrast, the rs2242480 variant has been identified in patients who respond to valproic acid therapy (Feng et al., 2018). Most patients included in the study were treated with valproic acid in combination with other pharmacotherapies, including CBD. Valproic acid was associated with adverse events in 8.5% of patients when administered with CBD (Table 2). This polymorphism has also been associated with response to other drugs, such as nervous-system depressants. The pharmacogenetics of this SNP was evaluated in a sample of 200 gynecological patients requiring general fentanyl analgesia. Patients with the Ho mut (AA) genotype responded better to fentanyl than those with the He (GA) or Ho wild-type (GG) genotype (Yan et al., 2018).

In a subsequent study involving 59 patients with gastric or intestinal cancer, the efficacy of opioid anesthesia during laparoscopic surgery was examined (oxycodone, n = 30; sufentanil, n = 29). The results indicated a more significant response to sufentanil in patients with the AA genotype of SNP rs2242480 than those with the GA+AA genotypes (p < 0.05), but this was not observed for oxycodone (Pu et al., 2019). In the current study, the focus was on adjuvant therapy with CBD in anti-seizure treatment. The presence of this genetic variant could be considered a risk factor for poor response to CBD treatment, which contradicts previous observations in a sample of patients with controlled and drug-resistant epilepsy (Feria-Romero et al., 2023). However, this tendency needs to be corroborated with more patients.

*CYP2B6* is the only gene in the human *CYP2B* subfamily that encodes a functional enzyme (Nebert et al., 2013). The *CYP2B6* consists of nine exons and is located on chromosome 19 at position 19q13.2. It is primarily expressed in the liver and catalyzes demethylation, hydroxylation, and oxidation to form active or inactive metabolites (Hidestrand et al., 2001; Ekins et al., 2008; Zhang et al., 2017). Substrates of CYP2B6 are found in ∼23 different therapeutic classes that are commonly used around the world.

When comparing the genetic variants with a Latin-American population of European descent (LA2) of rs8192709 and rs8192719, only the SNP rs8192709 had a significant difference (*p <* 0.0001) between the Alt allele (T) in the study population (0.4852) compared to LA2 (0.0256). Previous work found that the rate of the Alt allele (T) of this polymorphism was 7% in a Mexican pediatric population with solid embryonal tumors and poor response to ifosfamide treatment (Torres-Espindola et al., 2021), which is a lower rate than our results. Therefore, our results must be confirmed in the future with other molecular biology techniques. However, the clinical function of genetic variants in rs8192709 and rs8192719 has not been reported.

The CYP2D6 enzyme metabolizes about 20% of medications used in psychiatry, pain management, oncology, and cardiology (Taylor et al., 2020). Thus, pharmacogenetic consortia such as the Pharmacogenetic Guided Opioid Therapy advise against using these drugs for patients with specific genetic markers (Agullo et al., 2023). The SNP rs3892097 identifies the non-functional CYP2D6 *4 allele, which is linked to poor drug response, adverse effects, and comorbidities, although it was present in only 14% of patients in the CBD^pos^ group. Moreover, it has been described that CBD can inhibit various CYP enzymes, leading to potentially significant drug interactions, particularly for ASM (Brown and Winterstein, 2019; Landmark and Brandl, 2020).

CBD may notably inhibit CYP2C9, CYP2D6, and CYP2B6 (Bornheim et al., 1993; Jiang et al., 2013; Yamaori et al., 2010; Zendulka et al., 2016). Genetic alterations in these cytochromes directly affect the pharmacokinetics and metabolism of antiepileptic drugs (Kobayashi et al., 1999; Tan et al., 2010). Identifying altered cytochromes is particularly relevant for patients with DRE since these proteins are present in the blood–brain barrier and could affect drug metabolism directly at the target site (Ghosh et al., 2010; 2011).

The most important limitation of this study was the sample size. One of the reasons for the small sample size is the cost of commercial CBD (5,000 USD per year). Not all patients can afford it, and some use homemade preparations, in which the concentration of CBD is unknown. This limits the opportunity to meet the inclusion criteria of this work. In addition, although its medicinal use has been authorized, in public health programs, through which we recruited the patients, it is not included in the anti-seizure medications listed in the basic table for the treatment of epilepsy. Another limitation of this study was the lack of identification of the variant without CYP2C19*2 enzymatic activity in this population sample, which is discriminated by the detection of the SNP rs4244285. The reason was that in the analysis of polymorphisms by massive sequencing of a previous sample, we observed a lower frequency of the rs4244285 polymorphism in patients with drug-resistant epilepsy (0.16), compared to patients with controlled epilepsy (0.25). On the other hand, variants *3, *8, *9, and *10 without enzymatic activity and identified by polymorphisms rs4986893, rs41291556, rs17884712, and rs6413438, were not present in the previous population sample used as reference. Finally, the absence of the CYP2D6 enzyme known as the *5 variant or copies of *CYP2D6* variants (xN) was also not evaluated in this work, which should be analyzed in a future study using other technologies.

In conclusion, treatment with CBD reduces seizures in most patients by 68%, regardless of the cause or type of seizure. The rs8192709 SNP may be a genetic contributor to drug resistance, but this finding needs to be confirmed through additional molecular biology techniques, given its high prevalence compared to other populations. The *CYP3A4* *1/rs2242480 genotype was linked to a poor response to CBD in patients with DRE, which may be due to a stronger affinity between this enzyme and CBD, leading to its inactivation. This SNP is particularly relevant due to a higher occurrence in cases of DRE that do not respond to CBD in combination with ASM.

## Data Availability

The original contributions presented in the study are included in the article/Supplementary Material, further inquiries can be directed to the corresponding author.

## Abbreviations

7-COOH-CBD, 7-carboxy-cannabidiol; 7-OH-CBD, 7-hydroxy-cannabidiol; 6α-OH-CBD, 6-alpha-hydroxy-cannabidiol; ASM, Antiseizure medications; 6β-OH-CBD, 6-betha-hydroxy-cannabidiol; CBD, Cannabidiol; CBD^neg^, DRE patients who did not decrease their seizure frequency with CBD concomitant treatment; CBD^pos^, DRE patients who decreased their seizure frequency with CBD concomitant treatment; CI95%, Confidence Interval of 95%; CYP450, cytochromes P450; DRE, Drug-resistant epilepsy; FDA, Food and Drug Administration; 7-OH-CBD, 7-hydroxy-Cannabidiol; 7-COOH-CBD, 7-carboxy-Cannabidiol; LA2, Latin American population with European ancestry; MRI, Magnetic Resonance Imaging; NS, Not statistically significant; OR, Odds Ratio; SNP, Single nucleotide polymorphism; THC, Tetrahydrocannabinol; UGT, UDP-glucuronosyltransferase; M-W U, Mann -Whitney U.
